# Combination of Multiple Resistance Traits from Wild Relative Species in Chrysanthemum via Trigeneric Hybridization

**DOI:** 10.1371/journal.pone.0044337

**Published:** 2012-08-30

**Authors:** Yanming Deng, Jiafu Jiang, Sumei Chen, Nianjun Teng, Aiping Song, Zhiyong Guan, Weimin Fang, Fadi Chen

**Affiliations:** 1 College of Horticulture, Nanjing Agricultural University, Nanjing, Jiangsu, China; 2 Provincial Key Laboratory of Agrobiology, Jiangsu Academy of Agricultural Sciences, Nanjing, Jiangsu, China; United States Department of Agriculture, United States of America

## Abstract

**Background:**

With the objective of combining multiple resistant traits from wild relative species in florist’s chrysanthemums, trigeneric hybridization was conducted by crossing two intergeneric F_1_ hybrids *Chrysanthemum grandiflorum* × *Artemisia vulgaris* and *Chrysanthemum crassum* × *Crossostephium chinense*.

**Methodology/Principal Findings:**

To assess post-pollination phenomena, we investigated pollen germination on the stigma and embryo development, using fluorescence and scanning electron microscopy and paraffin-embedded sections, respectively. We selected eight putative trigeneric hybrid lines that showed the greatest morphological differences from the parents from among the progeny derived via embryo rescue. The hybridity of one trigeneric hybrid was further confirmed by fluorescent genomic *in situ* hybridization; in addition, the aphid resistance and salt tolerance of this hybrid were higher than those of the chrysanthemum parent and the *C. grandiflorum* × *A. vulgaris* F_1_ hybrid, respectively.

**Conclusions/Significance:**

The enhanced aphid resistance of the hybrid line reflects the inheritance of chromosomes from *A. vulgaris*, which carries genes that encode bioactive components. The enhanced salt tolerance of the trigeneric hybrid is attributable to inheritance of genetic materials from *Chrysanthemum crassum* and *Crossostephium chinense*, which act to maintain the compartmentation of Na^+^ and K^+^ ions and their selective transportation among different organs to avert deleterious effects and protect the photosynthetic apparatus. The results indicate that trigeneric hybridization between different bigeneric hybrids is a promising method for combination of multiple stress-resistance traits for improvement of chrysanthemum.

## Introduction

The tribe Anthemideae contains 12 subtribes, 108 genera and 1,741 species, of which about 30 genera are distributed in East Asia and 27 are present in China, including *Chrysanthemum* L. [1]. On the basis of the gene pool concept of Harlan and de Wet, chrysanthemum genetic resources are categorized into three groups in which the primary and secondary gene pools consist of the core species and closely related species, which are completely or partially cross-compatible with chrysanthemum [2], [3]. Garden chrysanthemum (*Chrysanthemum grandiflorum* (Ramat.) Tzvel.) has been cultivated for more than 1,600 years and is a popular cut and pot flower worldwide [4]. The important traits of some wild *Chrysanthemum* species have been incorporated into the gene pool of cultivated chrysanthemums, and reproductive barriers have been overcome successfully to improve chrysanthemum resistance traits through intergeneric hybridization [5]–[8]. Given the current trend in the horticultural industry for environment-friendly crop production, it is necessary to attempt to transfer additional useful genes from heterogeneric wild relative species to commercial chrysanthemum cultivars to raise novel genotypes that possess multi-resistance characteristics.

One of the most promising approaches by which to obtain multi-resistant genetic resources is the exploitation of multigeneric hybridization in breeding, involving species from different genera [9], [10]. Multigeneric hybrids (incorporating germplasm from three or more genera) may enable the transfer of different alien genes to a cultivated crop, and help to establish evolutionary relationships among different genomes included in the same genetic background [10]–[12]. However, in contrast to the many bigeneric hybrids recorded, reports of trigeneric hybrids are much fewer. At present, trigeneric hybridization has been successful only among a small number of species and mainly within the tribe Triticeae [9]–[14]. With regard to trigeneric hybrids among horticultural crops, only a few cut-flower vandaceous orchids [15] and citrangequat [11] are utilized for commercial usage.

*Chrysanthemum crassum* Kitamura, *Artemisia vulgaris* L. and *Crossostephium chinense* Makino are all members of the Anthemideae and are classified into either the primary or secondary gene pools of *Chrysanthemum sensu lato* [1], [2]. *Chrysanthemum crassum* is tolerant to salt stress and *A. vulgaris* is extremely resistant to insect and disease attack on account of bioactive components present in the essential oil [5], [16], [17]. *C. crassum* is tolerant to salt stress and *A. vulgaris* is extremely resistant to insect and disease for containing bioactive components in essential oil [5], [16], [17]. *Crossostephium chinense* has ornamental leaves with dense white tomentum and exhibits high levels of salt tolerance and pest resistance [18], [19]. Although severe reproductive barriers usually hinder intergeneric hybridization of chrysanthemum [5], [6], intergeneric hybrids between *C. crassum* and *C. chinense* [19], and between *C. grandiflorum* and *A. vulgaris* [5], have been obtained successfully via embryo rescue and have given rise to many chrysanthemum intergeneric hybrids.

As a novel genetic resource, the intergeneric hybrid *C. grandiflorum* × *A. vulgaris* not only blooms normally, but also shows enhanced resistance to chrysanthemum aphids and Alternaria leaf spot, as well as superior rooting ability than its chrysanthemum parent [5], [8]. To further expand the gene pool, transfer desirable genes from *C. chinense* and *C. crassum* to chrysanthemum cultivars, and create new multi-resistant germplasm, we performed an artificial cross between two bigeneric F_1_ hybrids, namely *C. grandiflorum* × *A. vulgaris* (female parent, hereafter CA) and *C. crassum* × *C. chinense* F_1_ (male parent, hereafter CC), to which embryo rescue was applied to obtain progeny. Previous reports on trigeneric hybrids mainly focused on either the production and identification, or study of the morphology and cytogenetics, of the hybrids [9], [10], [12]–[14]. Few investigations have examined directly reproductive characteristics of a specific cross, such as pollen–pistil interaction and hybrid embryo development, nor the combination of resistance to biotic and/or abiotic stresses.

Pollen–pistil interaction and embryo development, which represent pre- and post-fertilization stages, respectively, may indicate the crossability and genetic compatibility of the parents [6], [20], [21]. Thus in the present study we systematically investigated these aspects to provide basic scientific information on the cross-compatibility of the two bigeneric hybrids. In addition, we compared the aphid resistance and salt tolerance of the progeny with their parents to determine the effectiveness of trigeneric hybridization for combination of chrysanthemum resistance traits.

## Results

### Pollen-pistil Interaction of CA×CC

**Figure 1:**
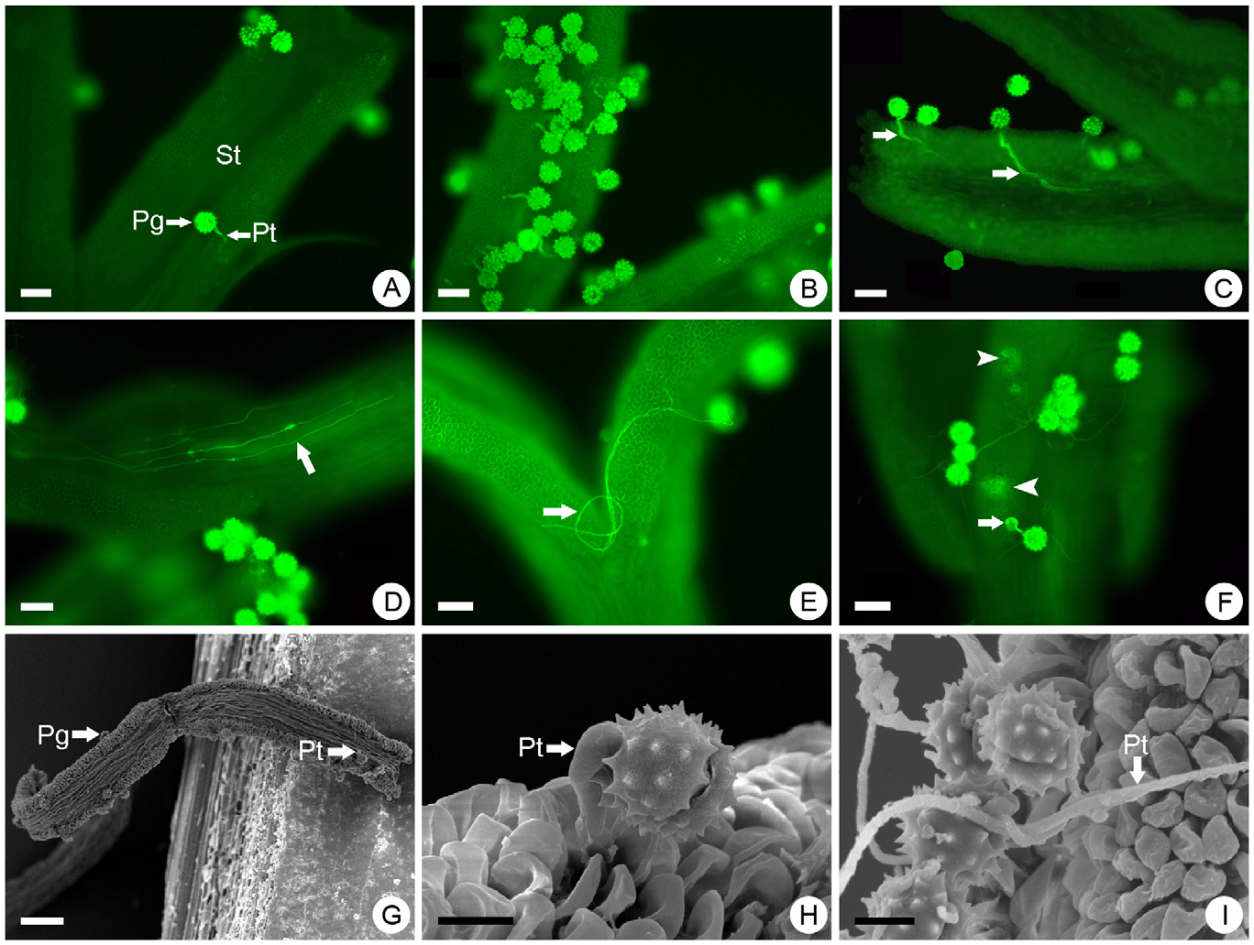
Pollen germination and pollen tube growth of F_1_ *C. crassum* × *C. chinense* on the stigma of F_1_ *C. grandiflorum* ‘Zhongshanjingui’ × *A. vulgaris* ‘Variegata’ plants. (A) Germinated pollen grains on the stigma 1 hour after pollination (HAP). (B) At 2 HAP, a large number of pollen grains had germinated and many pollen tubes had penetrated the stigma. (C) At 4 HAP, the pollen tubes were growing toward the style (marked by arrows). (D) At 12 HAP, some pollen tubes were growing in the style (indicated by arrow). (E) At 24 HAP, abnormal (twisted and coiled) pollen tubes on the stigma (indicated by arrow). (F) At 48 HAP, swollen pollen tube tip (arrow), twisted tubes and callose deposition (arrow heads) on the stigmatic surface were observed. (G–I) Scanning electron micrographs: (G) pollen grains adhering to the stigma at 1 HAP, (H) short pollen tube penetrating the stigma at 2 HAP, (I) twisted and extended pollen tubes at 24 HAP. Abbreviations: Pg, pollen grain; Pt, pollen tube; St, stigma. Bars: a–f: 50 µm; g: 100 µm; h–i: 10 µm.

At 1 hour after pollination (HAP), ∼10 pollen grains adhered to each pistil and some pollen tubes were observed to have penetrated into the stigma (Fig. 1A). At 2 HAP, the number of pollen grains and pollen tubes on the stigma both increased (∼30 grains on average) (Fig. 1B). Subsequently, pollen tubes growing towards the style within the stigmatic tissues were observed at 4 HAP (Fig. 1C) and were growing along the style at 12 HAP (Fig. 1D). Abnormal growth of some pollen tubes was observed at 24 HAP, for example, coiling of the tube on the stigma surface (Fig. 1E). The germination of pollen grains occurred up to 48 HAP, but additional abnormalities (tube tip swollen, and tube extended or twisted) and callose deposition were observed at that time (Fig. 1F). Scanning electron micrographs showed similar results, that is, pollen grains germinated within 1 HAP (Fig. 1G), short pollen tubes penetrated clearly into the stigma at 2 HAP (Fig. 1H), and many tubes showed abnormalities at 24 HAP (Fig. 1I).

### Embryo, Endosperm and Embryo Sac Development of CA × CC

**Figure 2:**
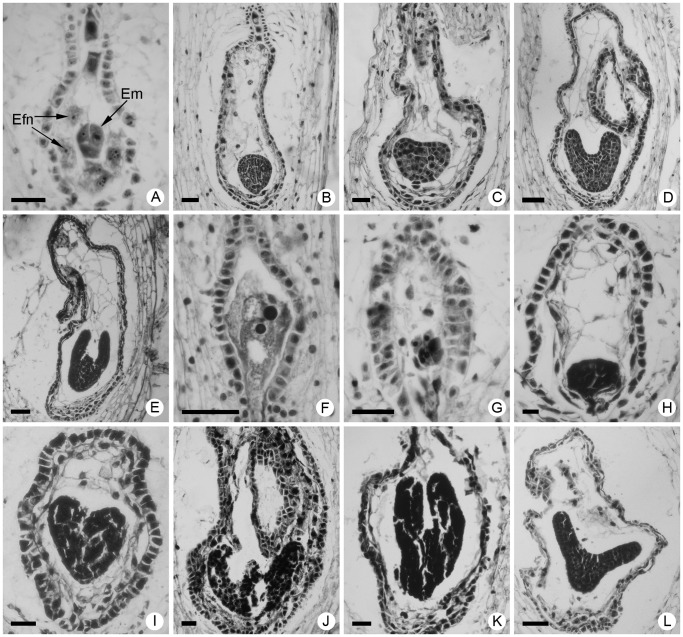
Development of hybrid embryos in the cross between F_1_ *C. grandiflorum* ‘Zhongshanjingui’ × *A. vulgaris* ‘Variegata’ (♀) and F_1_ *C. crassum* × *C. chinense* (♂). (A) Multicelled proembryo and free nuclei of endosperm at 2 days after pollination (DAP). (B) Globular proembryo and free nuclear endosperm at 4 DAP. (C) Early heart-shaped embryo at 6 DAP; note the crimpled integumentary endothelium and reduced number of endosperm nuclei. (D) Torpedo-shaped embryo at 8 DAP. (E) Cotyledon-shaped embryo at 10 DAP; the endosperm is almost eliminated. (F) An unfertilized embryo sac at 4 DAP. (G) Degenerated multicelled proembryo. (H) Degenerated globular proembryo. (I) Degenerated heart-shaped embryo. (J) Degenerated torpedo-shaped embryo. (K) Degenerated cotyledon-shaped embryo. (I) An abnormal cotyledon-shaped embryo; note the bilateral cotyledons elongated laterally rather than along the longitudinal axis. Abbreviations: Efn: endosperm free nucleus; Em: embryo. Scale bars: a–c and g–j: 40 µm; d–f and k–l: 80 µm.

The zygote had undergone several mitotic divisions and developed into a multicelled proembryo at 2 days after pollination (DAP) (Fig. 2A). At this time point, the endosperm consisted of free nuclei and the embryo sac was bottle shaped (Fig. 2A). By 4 DAP, 24.5% of the proembryos were globular in shape and the embryo proper was about 55 µm in diameter (Table 1; Fig. 2B); the endosperm had proliferated to about 10 nuclei within the embryo sac, which had elongated to about 350 µm in length, thus quintupling the size of the embryo (Table 1; Fig. 2B). A further 2 d later, 22.9% of the embryos had developed to the heart-shaped stage and were about 125 µm in length and 90 µm in width (Table 1; Fig. 2C). Endosperm cellularization had occurred with formation of a cell wall around each nucleus, and the endothelium was about 450 µm in length and 220 µm in width (Table 1; Fig. 2C). By 8 and 10 DAP, 16.8% and 14.4% of the embryos were torpedo- and cotyledon-shaped, about 230 µm and 290 µm in length, and 195 µm and 230 µm in width, respectively, and showed an obvious elongation in the region of the cotyledons, which were clearly recognizable (Table 1; Fig. 2D, E). The endosperm was almost eliminated, and the endothelium cells had expanded into the nucellus and into the external space within the embryo sac with the tissue crimpling further (Fig. 2D, E).

**Table 1:** Development of embryos at 4–12 days after pollination (DAP) in the cross of (*Chrysanthemum grandiflorum* × *Artemisia vulgaris*) F_1_ × (*C. crassum* × *Crossostephium chinense*) F_1_.

									Size of normal embryo			
			Normalembryos		Abortedembryos		Totalembryos		Embryo body		Embryosac	
DAP	Development stage	No. of ovules	No.	Rate (%)	No.	Rate (%)	No.	Rate (%)	Length (µm)	Width (µm)	Length (µm)	Width (µm)
4	Globular	110	27	24.5	35	31.8	62	56.4	70	55	350	130
6	Heart	105	24	22.9	32	30.5	56	53.3	125	90	450	220
8	Torpedo	101	17	16.8	34	34.8	51	50.5	230	195	720	255
10	Cotyledon	104	15	14.4	43	41.3	58	55.8	290	210	830	270
12	Maturing	112	16	14.3	35	37.3	51	45.5	380	230	870	305
Total	–	532	99	18.6	179	33.6	278	52.3	–	–	–	–

About 10% of the ovules were unfertilized until 4 DAP (Fig. 2F). Hybrid embryos aborted at various stages, from the multicelled proembryo (Fig. 2G) to globular (Fig. 2H), heart-shaped (Fig. 2I), torpedo-shaped (Fig. 2J) and cotyledon-shaped embryo stages (Fig. 2K). Sometimes, the two developing cotyledons elongated laterally rather than along the longitudinal axis (Fig. 2L). In contrast to the sharply decreased frequency of normal embryos, the embryo abortion frequency rose slowly from the lowest level of 30.5% at 6 DAP (i.e., the heart-shaped stage) to the highest frequency of 41.3% at 10 DAP (i.e., the cotyledon-shaped stage) (Table 1). Overall, the embryo abortion frequency was 33.6% of the ovules observed from 4 to 12 DAP (Table 1).

### Embryo Rescue and Morphological Characteristics of the Putative Trigeneric Hybrids

Of the 400 rescued embryos obtained from plump ovaries, 103 germinated and survived in the greenhouse. Ninety-five lines grew to maturity and flowered normally in the field. It is very laborious to evaluate either aphid resistance or salinity tolerance of each line with molecular cytogenetic methods and, more importantly, the aim of this study was to obtain trigeneric hybrids with improved multi-resistance to provide germplasm resources for future breeding. Thus, we first carried out a morphological investigation for preliminary screening of putative trigeneric hybrids from among the progeny that showed the greatest differences from the parents. After preliminary identification of morphological characteristics at stages of flowering, eight putative hybrid lines (hereafter referred to as T_1_, T_2_, T_3_, T_4_, T_5_, T_6_, T_7_ and T_8_, respectively) that showed the greatest differences from the parents were selected for further study.

**Figure 3:**
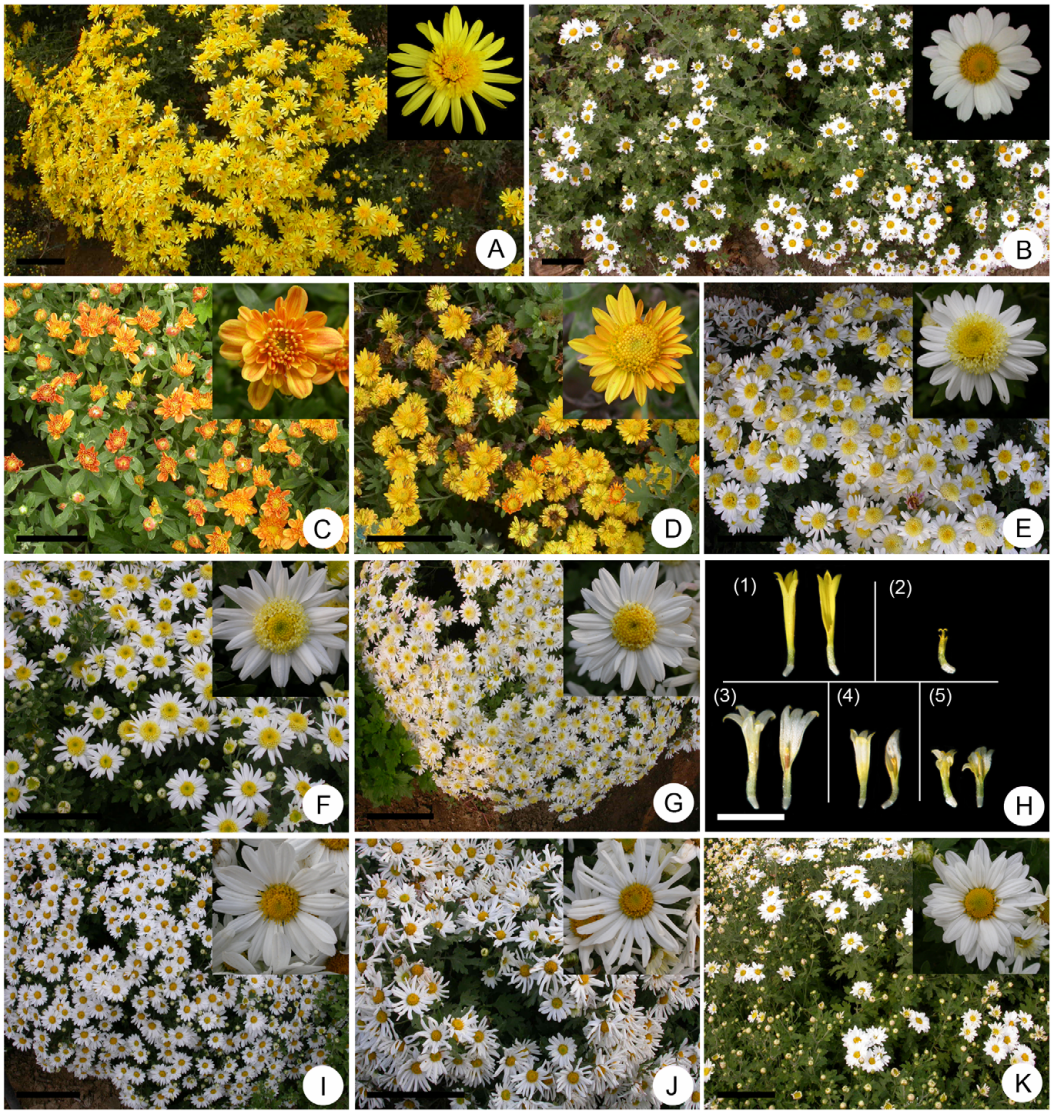
Floral morphology of trigeneric hybrid plants between F_1_ *C. grandiflorum* ‘Zhongshanjingui’ × *A. vulgaris* ‘Variegata’ (♀) and F_1_ *C. crassum* × *C. chinense* (♂). (A) Standard anemone type and yellow inflorescences of the maternal parent. (B) Non-anemone type and white inflorescences of the paternal parent. (C–K) Putative trigeneric hybrid plants (C: T_1_, double type and nacarat flower; D: T_2_, single type and yellow flower; E: T_3_, standard anemone type and white flower; F: T_4_, less clear anemone type and white flower; G: T_5_, least clear anemone type and white flower; H: tubular florets of the female parent (1), male parent (2), and the hybrid lines T_3_ (3), T_4_ (4) and T_5_ (5), respectively; note the pistil in the florets; I: the hybrid line T_6_, single type and white flower with closely set ray florets; J: the hybrid line T_7_, single type and white flower with ray florets more widely spaced; K: the hybrid line T_8_, double type and white flower with 2–3 layers of ray florets). Scale bars: a–g and i–k: 10 cm; h: 10 mm.

Although the dates of onset of flowering for the maternal and paternal plants were similar (8 October and 13 October, respectively), the onset of flowering among the eight progeny varied by 35 d between the earliest and latest dates (20 September and 25 October, respectively; Table 2). The average plant height and crown width of the maternal parent were 48.8 and 66.2 cm, respectively, and those of the paternal parent were 60.6 and 90.4 cm, respectively (Table 2). Among the eight hybrid plants these two traits differed significantly from both their parents and among the progeny; the shortest hybrid was shorter than the maternal parent and the tallest hybrid was taller than the paternal parent (Table 2). All hybrid progeny differed from the parents in flower and leaf morphological traits. The respective inflorescence type of the maternal parent CA and paternal parent CC was standard anemone (Fig. 3A) and non-anemone single-petal (Fig. 3B). The hybrid progeny exhibited six different inflorescence types, namely three different degrees of anemone types (standard, less clear and least clear anemone) and three different non-anemone petal types (single-, double- and multi-petal) (Table 2; Fig. 3C–K). The inflorescence colour of CA and CC was yellow and white, respectively, whereas the T_1_ and T_2_ progenies expressed novel nacarat and orange colours (Table 2; Fig. 3). Other investigated flower and leaf traits, comprising leaf length and width, capitulum and disc diameters, and numbers of ray and tubular florets, all differed significantly from their parents (Table 2). Thus the hybridity of the eight progeny was initially confirmed on the basis of the morphological traits analysed.

**Table 2:** Morphological traits of *Chrysanthemum grandiflorum* × *Artemisia vulgaris* F_1_ (CA, ♀), *C. crassum* × *Crossostephium chinense* F_1_ (CC, ♂) and their putative hybrid lines.

Plant lines	Dates of flowering (day/month)	Type^a^ and color^b^ of flower	Plant height (cm)	Crown width (cm)	Leaf characteristics			Inflorescence characteristics^c^					
					Length (cm)	Width (cm)	Length/Width	DD (cm)	ID (cm)	DD/ID	NT	NL	NT/NL
CA	08/10	A1 and Y	48.8^B^ *	66.2	5.96±0.39*	3.56±0.33	1.68±0.15	2.37±0.22A	5.24±0.10	0.45±0.04C	114.2±9.6	21.5±1.8	5.4±0.8
CC	13/10	S and W	60.6	90.4	5.85±0.34	4.05±0.22	1.45±0.08	1.50±0.07	4.74±0.20	0.32±0.02	170.8±8.4	22.6±2.2	7.6±0.7
T_1_	20/09	M and N	31.6	37.4	4.90±0.63	2.61±0.38	1.89±0.21	0.61±0.09	3.48±0.24	0.18±0.03	39.5±5.8	125.4±6.5	0.3±0.1
T_2_	25/10	S and O	33.9	34.7	4.36±0.30	2.85±0.15	1.53±0.09	1.46±0.10	3.27±0.18	0.45±0.03	161.2±8.1	29.0±2.2	5.6±0.5
T_3_	08/10	A1 and W	47.5	65.8	8.20±0.45	4.19±0.27	1.96±0.08	1.76±0.10	4.20±0.21	0.42±0.03	174.7±5.4	21.4±0.8	8.2±0.4
T_4_	08/10	A2 and W	39.3	55.5	4.49±0.31	2.73±0.27	1.66±0.18	1.65±0.11	3.88±0.27	0.43±0.03	116.6±7.0	19.0±1.5	6.2±0.6
T_5_	12/10	A3 and W	48.2	73.3	6.61±0.60	3.93±0.29	1.69±0.14	1.59±0.15	4.22±0.24	0.38±0.02	104.4±6.1	32.6±2.1	3.2±0.2
T_6_	02/10	S and W	61.2	92.7	7.12±0.39	4.45±0.28	1.60±0.11	1.42±0.08	4.37±0.16	0.32±0.01	161.2±9.1	32.1±1.6	5.0±0.4
T_7_	16/10	S and W	46.2	79.1	6.33±0.60	3.76±0.30	1.69±0.12	1.26±0.05	4.43±0.18	0.28±0.02	109.9±9.9	23.7±1.7	4.7±0.6
T_8_	10/10	D and W	68.1	120.4	7.97±0.54	5.37±0.36	1.48±0.04	1.51±0.09	5.43±0.25	0.28±0.02	159.9±11.1	43.7±4.5	3.7±0.3

a A1, Standard anemone type; A2, Less clear anemone type; A3, Least clear anemone type; D, Double-petal type; M, Multi-petal type; S, Single-petal type;

b N, Nacarat; O, Orange; W, White; Y, Yellow;

c DD, central disc diameter; ID, inflorescence diameter; NT, number of tubular disc florets; NL, number of ligulate ray florets;

* The values represent the mean ± SD.

### Genomic *in situ* Hybridization

**Figure 4:**
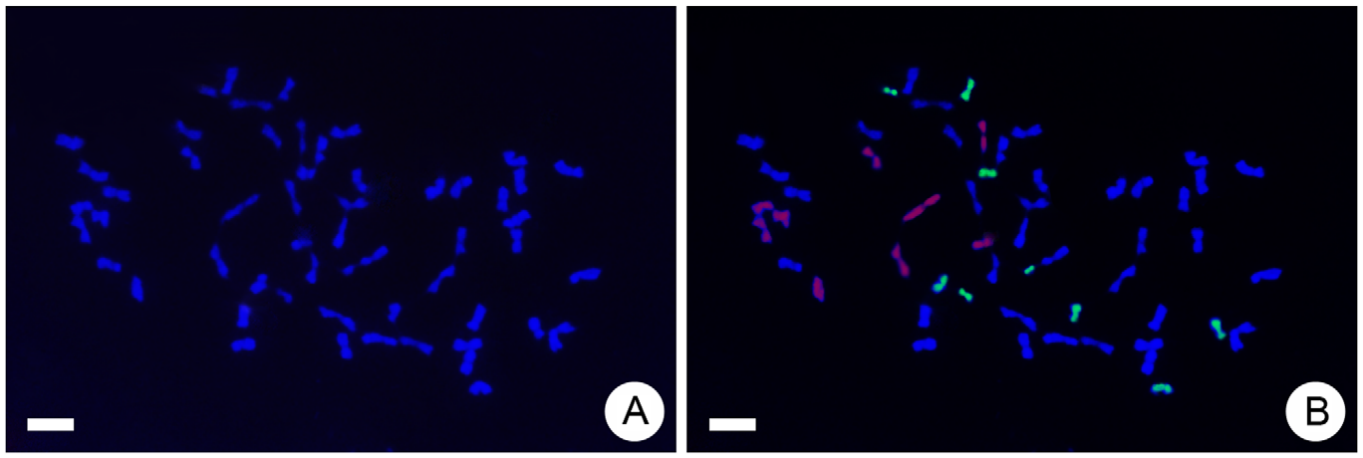
GISH analysis of the trigeneric hybrid line T_3_. (A) All 54 chromosomes showing blue fluorescence after staining with DAPI. (B) Among the 54 chromosomes, nine fluoresced green after staining with the probe for *Crossostephium chinense* genomic DNA, and nine fluoresced red after staining with the probe for *Artemisia vulgaris* genomic DNA; thus the other 36 chromosomes were obtained from *C. grandiflorum* ‘Zhongshanjingui’ and/or *C. crassum*. Bars: 5 µm.

Among the progeny, the line T_3_ (a hexaploid with 2*n* = 6*x* = 54) was analysed by fluorescent genomic *in situ* hybridization (GISH). Nine chromosomes fluoresced green with the *C. chinense* genomic DNA probe, and nine fluoresced red with the *A. vulgaris* genomic DNA probe (Fig. 4A, B). The other 36 chromosomes were obtained from *C. grandiflorum* ‘Zhongshanjingui’ and *C. crassum*, which are difficult to distinguish with GISH because of the close genetic relationship among *Chrysanthemum* species [22]. The T_3_ line was confirmed to be a true trigeneric hybrid containing chromosomes from *Chrysanthemum*, *Artemisia* and *Crossostephium*, thus the following resistance test was only conducted on the T_3_ hybrid.

### Aphid Resistance

At 21 d post-inoculation, the inoculated aphids had survived and multiplied on each test plant but their MR showed highly significant differences. The MR of the control ‘Zhongshanjingui’ and the paternal parent CC was high (9.1 and 8.3, respectively), thus both plants were classified as weakly resistant. In contrast, the maternal parent CA was highly resistant to aphids (MR 3.6; Table 3). The trigeneric hybrid T_3_ showed moderate resistance (MR 4.9; Table 3). The novel trigeneric hybrid showed slightly lower resistance to aphids than the maternal parent CA, but showed higher resistance than ‘Zhongshanjingui’ and the paternal parent CC. This difference was represented respectively by a negative IR (−8.3%) relative to CA and positive IRs (57.1 and 53%) relative to ‘Zhongshanjingui’ and CC, respectively (Table 3).

**Table 3:** Aphid resistance of *Chrysanthemum* ‘Zhongshanjingui’ (Zh), *C. grandiflorum* × *Artemisia vulgaris* F_1_ (CA, ♀), *C. crassum* × *Crossostephium chinense* F_1_ (CC, ♂) and their trigeneric hybrid line T_3_.

Plant lines	MR*	RG**	IR_Zh_ (%)***	IR_CA_ (%)	IR_CC_ (%)
Zh	9.1±1.1^a^	L	–	−152.8	−9.6
CA	3.6±0.3^c^	H	60.4	–	56.6
CC	8.3±0.7^b^	L	8.8	−130.6	–
T_3_	3.9±0.2^c^	H	57.1	−8.3	53.0

* MR, multiplication rate of aphids. Values represent mean ± SE, and different superscripts indicate significant differences at P<0.05 according to Duncan’s test.

** RG, resistance grade; L, lowly resistant; H, highly resistant.

*** IR_Zh_, IR_CA_ and IR_CC_, inhibition ratio relative to Zh, CA and CC. The calculated formula was shown in the section of Materials and methods. ‘-’ represented no value.

### Salt Tolerance

**Figure 5:**
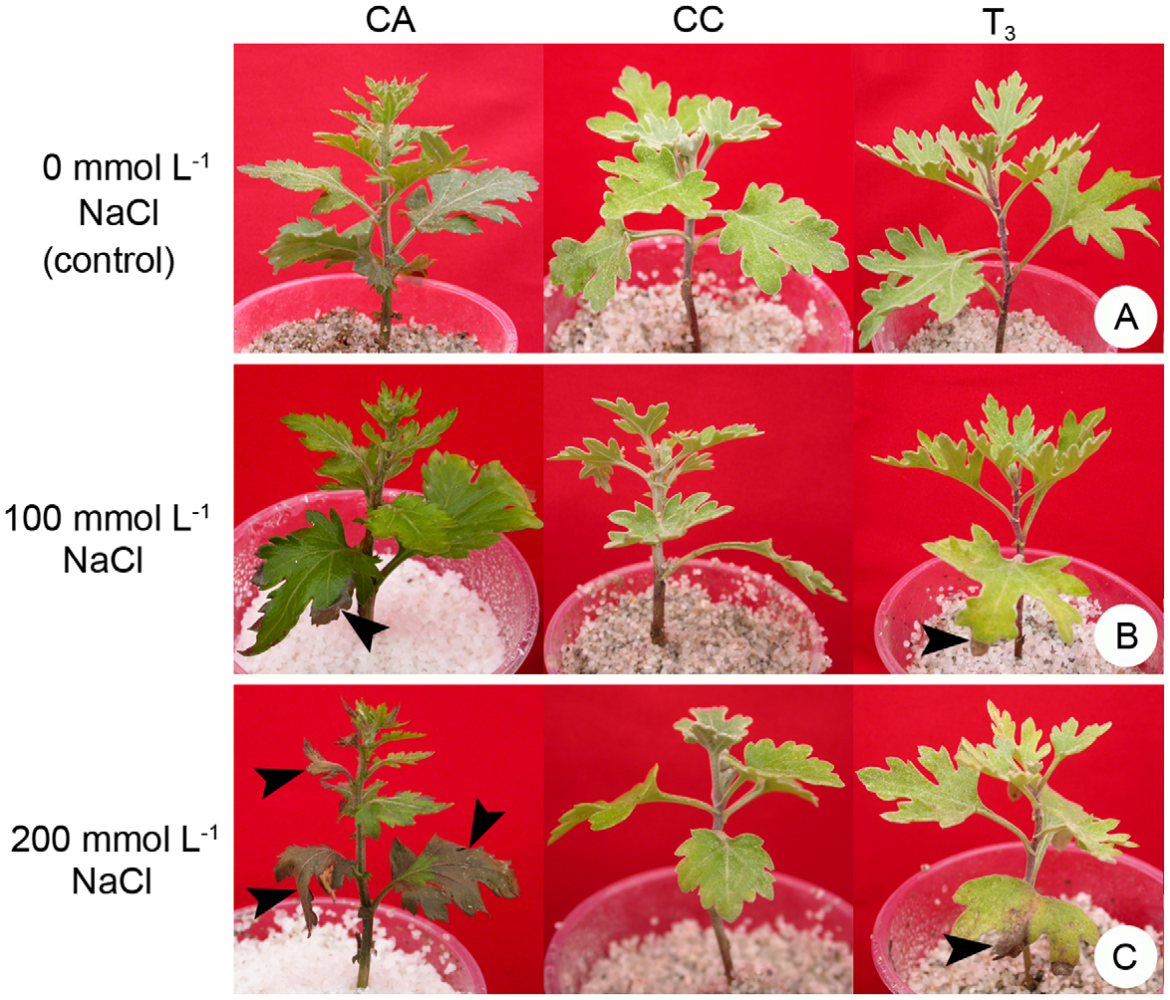
Salinity tolerance test on the trigeneric hybrid and the parents (F_1_ *C. grandiflorum* × *Artemisia vulgaris*, ♀, CA; and F_1_ *C. crassum* × *Crossostephium chinense*, ♂, CC). Plants were cultured in Hoagland solution supplemented with (A) 0, (B) 100 and (C) 200 mmol L^−1^ NaCl. Left to right: CA, CC and the hybrid line T_3_. The arrowheads in (B) and (C) indicate the damaged leaves.

After a 7-day adaptive period, the test plants grew normally in Hoagland solution without NaCl stress (Fig. 5A). However, the plants suffered different degrees of injury when cultured in saline solutions. In 100 mmol L^−1^ NaCl solution, the maternal parent CA and the trigeneric hybrids showed obvious symptoms of salinity injury, etiolation or necrosis of the leaf tips, whereas the paternal parent CC was normal (Fig. 5B). At 200 mmol L^−1^ NaCl, the paternal parent still showed no obvious abnormalities, whereas almost all leaves of the maternal parent were dead (Fig. 5C). The trigeneric hybrids also suffered more severe injury than the paternal parent, but the injury was much less severe than the maternal parent and was mainly restricted to the lowest leaves (Fig. 5C). Overall, the trigeneric hybrids showed higher salt tolerance than that of the maternal parent and lower tolerance than that of the paternal parent (Fig. 5).

### Ultrastructure of Mesophyll Cells Under Salt Stress

**Figure 6:**
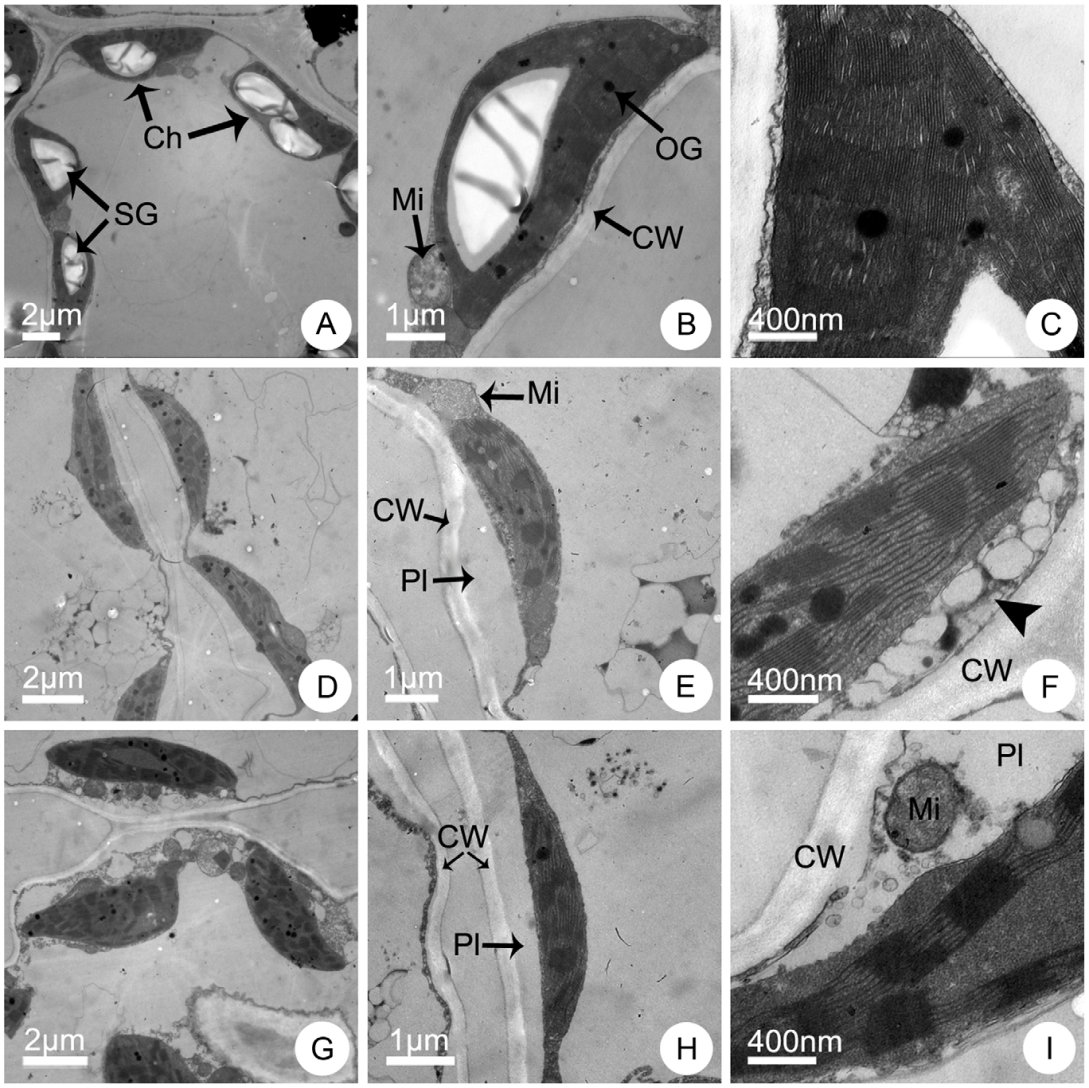
Ultrastructural observation of mature leaves of the maternal parent F_1_ *C. grandiflorum* × *A. vulgaris* (CA) after one week of NaCl stress. Leaf ultrastructure of plants cultured in Hoagland solution supplemented with (A–C) 0 (control), (D–F) 100 and (G–I) 200 mmol L^−1^ NaCl. Ch, chloroplast; CW, cell wall; Mi, mitochondria; OG, osmiophilic globules; Pl, plasmolysis; SG, starch grains. The arrowhead in (F) indicates the vesicles.

**Figure 7:**
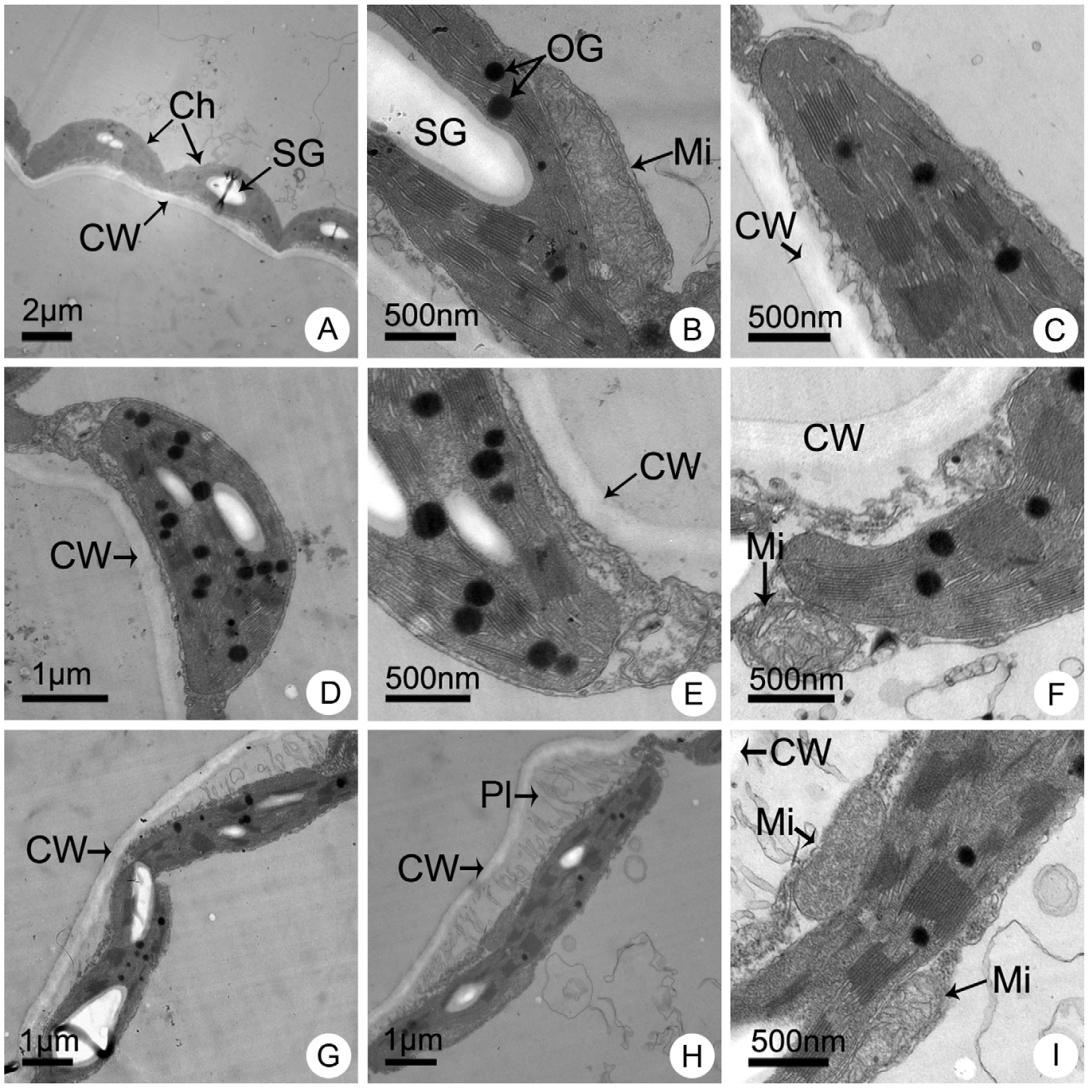
Ultrastructural observation of mature leaves of the paternal parent F_1_ *C. crassum* × *C. chinense* (CC) after one week of NaCl stress. Leaf ultrastructure of plants cultured in Hoagland solution supplemented with (A–C) 0 (control), (D–F) 100 and (G–I) 200 mmol L^−1^ NaCl. Ch, chloroplast; CW, cell wall; Mi, mitochondria; OG, osmiophilic globules; Pl, plasmolysis; SG, starch grains.

**Figure 8:**
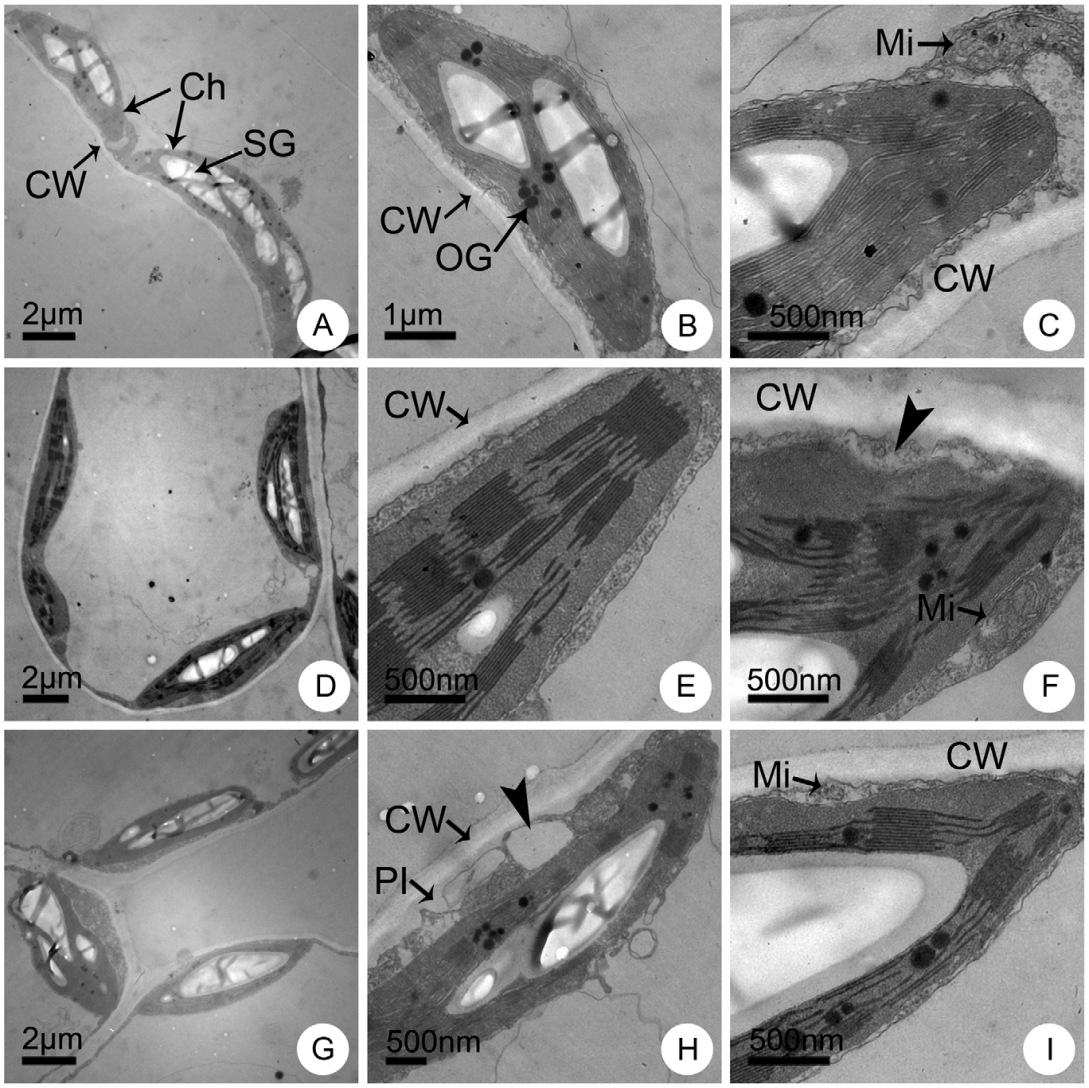
Ultrastructural observation of mature leaves of the trigeneric hybrid line T_3_ CA × CC after one week of NaCl stress. Leaf ultrastructure of plants cultured in Hoagland solution supplemented with (A–C) 0 (control), (D–F) 100 and (G–I) 200 mmol L^−1^ NaCl. Ch, chloroplast; CW, cell wall; Mi, mitochondria; OG, osmiophilic globules; Pl, plasmolysis; SG, starch grains. The arrowheads in (F) and (H) indicate the vesicles.

The mesophyll cells in leaves of normal appearance from different lines showed significantly different ultrastructural characteristics after 7 d of NaCl treatment. Without salinity stress, the mesophyll cells from different lines were of similar regular shapes with a smooth outline. The plasma membrane was in close contact with the wall, and the mitochondrial structure was normal. In the chloroplasts, the regular grana contained many lamellae and a large number of stacks were present (Fig. 6A–C; 7A–C; 8A–C). However, the cells showed distinct differences under 100 mmol L^−1^ NaCl treatment. The cells of the maternal parent CA suffered severe damage and were irregularly shaped (Fig. 6D). Some cells appeared plasmolysed and the outer membrane of the mitochondria was indistinct (Fig. 6E). The grana stacks were irregularly shaped and the quantity of stacks decreased significantly (Fig. 6F). In contrast, the cells of the paternal parent CC were almost normal except for only a slight degree of plasmolysis. The chloroplasts appeared to be better developed, were larger and contained a greater number of starch grains (Fig. 7D). The plasma membrane was in close contact with the cell wall, and the mitochondria, grana, grana lamellae and stacks were normally developed (Fig. 7E). Only a small portion of membranes of several cells showed slight plasmolysis, but the outer membrane of mitochondria was intact and the mitochondrial cristae were distinct (Fig. 7F). With regard to the trigeneric hybrid, the cells showed a similar ultrastructure to that of CC. The leaf mesophyll cell margins were regular and the number of starch grains showed almost no change, although the size of the starch grains decreased (Fig. 8D). The plasma membrane was in close contact with the cell wall, and the shapes of the mitochondria, grana, lamellae and stacks were normal (Fig. 8E). A small portion of membranes in several cells showed a slight degree of plasmolysis, but the outer mitochondrial membrane remained intact and the mitochondrial cristae were normal (Fig. 8F).

Under 200 mmol L^−1^ NaCl stress, the mesophyll cells showed a major difference. The cells of CA showed irregular margins (Fig. 6G) and more serious plasmolysis (Fig. 6H). The grana, lamellae and stacks were indistinct and reduced in number. The mitochondria were detached from chloroplasts and had a cracked or folded outer membrane and the cristae had disappeared (Fig. 6I). Although some mesophyll cells of CC also showed serious plasmolysis, the cell margins remained distinctly regular (Fig. 7G, H). However, a portion of the grana, lamellae and stacks were indistinct and decreased in number. The outer membrane of some mitochondria was cracked and the inner mitochondrial cristae were indistinct, but the mitochondria were still in close contact with chloroplasts (Fig. 7I). The mesophyll cell ultrastructure of the trigeneric hybrid was largely similar to that of CC. The cell margins remained largely regular (Fig. 8G) and some cells showed a slight degree of plasmolysis (Fig. 8H). A portion of the grana, lamellae and stacks were indistinct and decreased in number. The mitochondrial outer membrane was cracked and the inner cristae indistinct, but the mitochondria were in close contact with chloroplasts (Fig. 8I).

### Contents of Na^+^ and K^+^ Ions Under Salt Stress

Without additional NaCl, the maternal parent CA showed relatively low contents of Na^+^ and K^+^ ions. The content of Na^+^ was especially low and consequently the K^+^/Na^+^ ratio was high. The distribution of K^+^ ions differed among organs because of the lower transport from roots to stems (TS_K,Na_ 0.57) and higher transport from stems to leaves (TS_K,Na_ 2.34) (Table 4). The content of Na^+^ increased with the elevation in NaCl concentration, especially in the leaf, which showed a rapid increase at a NaCl concentration exceeding 100 mmol L^−1^ (Table 4). Generally, under low salinity treatment, the Na^+^ content was always highest in the leaf, followed by the stem and lowest in the root. Under high salinity treatment (200 mmol L^−1^ NaCl), however, the Na^+^ content in the stem increased rapidly and was about three-fold that of the root and ten-fold that of the control (Table 4).

**Table 4:** Comparison of Na^+^ and K^+^ accumulation in different organs and transport in the parents *Chrysanthemum grandiflorum* × *Artemisia vulgaris* F_1_ (CA, ♀) and *C. crassum* × *Crossostephium chinense* F_1_ (CC, ♂), and their trigeneric hybrid T_3_ treated with different NaCl concentrations.

Plant lines	Added NaCl (mmol L^−1^)	Organs^a^	Ion content (mg g^−1^ DW)^b^				TS_K, Na_ c
			Na^+^	K^+^	Na^+^ + K^+^	K^+^/Na^+^	
CA	0	R	3.17±0.07	31.66±1.46	34.83±1.53	9.97±0.24	–
		S	2.16±0.05	12.21±0.31	14.38±0.35	5.64±0.04	0.57±0.01
		L	1.95±0.03	25.72±1.28	27.67±1.31	13.19±0.45	2.34±0.07
	50	R	5.88±0.22	26.21±1.59	32.09±1.81	4.45±0.11	–
		S	4.82±0.11	10.89±1.04	15.71±1.15	2.26±0.16	0.51±0.02
		L	9.46±0.29	19.29±0.76	28.74±1.05	2.04±0.02	0.91±0.06
	100	R	7.05±0.20	25.78±2.13	32.83±2.33	3.65±0.20	–
		S	6.70±0.29	9.98±0.86	16.68±1.14	1.49±0.06	0.41±0.00
		L	28.25±2.09	18.16±1.12	46.40±3.21	0.64±0.01	0.43±0.02
	150	R	8.40±0.43	25.04±1.36	33.45±1.79	2.98±0.01	–
		S	7.25±0.31	8.38±0.36	15.64±0.67	1.16±0.01	0.39±0.00
		L	33.92±2.04	20.53±1.28	54.45±3.32	0.61±0.00	0.52±0.00
	200	R	6.78±0.54	21.55±1.66	28.33±2.19	3.18±0.01	–
		S	21.15±1.71	13.68±0.91	34.83±2.61	0.65±0.01	0.20±0.00
		L	40.04±2.26	20.84±1.84	60.88±4.10	0.52±0.02	0.80±0.04
CC	0	R	2.70±0.11	32.06±2.68	34.76±2.77	11.88±0.72	–
		S	1.19±0.11	17.73±1.00	18.92±1.10	14.94±0.70	1.26±0.10
		L	4.33±0.29	29.84±1.89	34.17±2.18	6.89±0.03	0.46±0.02
	50	R	7.30±0.30	32.39±1.81	39.69±2.11	4.43±0.07	–
		S	5.55±0.21	19.05±1.04	24.60±1.25	3.43±0.06	0.77±0.01
		L	11.99±1.02	30.61±1.69	42.59±2.69	2.56±0.08	0.75±0.03
	100	R	9.24±0.83	32.60±2.00	41.84±2.82	3.53±0.10	–
		S	5.44±0.29	20.58±1.75	26.03±2.04	3.78±0.12	1.07±0.06
		L	18.20±1.24	27.18±1.98	45.38±3.22	1.49±0.01	0.40±0.01
	150	R	10.53±0.70	28.44±1.49	38.97±2.18	2.70±0.04	–
		S	5.47±0.29	16.76±1.18	22.23±1.47	3.06±0.05	1.13±0.04
		L	18.51±1.70	34.66±2.13	53.17±3.83	1.88±0.06	0.61±0.03
	200	R	12.19±1.19	29.40±1.56	41.59±2.72	2.42±0.13	–
		S	4.50±0.32	16.79±0.82	21.29±1.13	3.74±0.08	1.55±0.05
		L	19.72±1.52	37.12±1.90	56.83±3.42	1.88±0.05	0.50±0.00

a R, root; S, stem; L, leaf;

b the values represent mean ± SD; − represents no value;

c S_K, Na_ represents the selectivity ratio of K^+^ and Na^+^; TS_K, Na_ (transportation S_K, Na_) = (the value K^+^/Na^+^ of sink organ)/(the value K^+^/Na^+^ of source organ).

The paternal parent CC showed a significant difference to CA. The Na^+^ content was always highest in the leaf, followed by the root and lowest in the stem under all treatments (Table 4). The Na^+^ content in the root was always higher, and that in the leaf was always lower, than those of CC, under high salinity (NaCl concentration exceeding 50 mmol L^−1^) (Table 4). However, the relative trends differed at 200 mmol L^−1^ NaCl, under which the leaf Na^+^ content in CC was less than half that of CA. The values for TS_K,Na_ from roots to the stem were always >1, whereas those from the stem to the leaf were <1 (Table 4).

The rank order of Na^+^ content of the trigeneric hybrid in different organs was leaf > stem > root. Although the hybrid exhibited relatively higher Na^+^ contents in different organs than those of the male parent, the Na^+^ and K^+^ contents were always significantly lower than those of the female parent (Table 5). The K^+^/Na^+^ ratios were always higher than those of CA and equivalent to those of CC. Overall, the trigeneric hybrid showed a similar TS_K,Na_ value to CA (<1). However, the TS_K,Na_ value for the leaf was higher than that of the stem, which was opposite to that of CA, but similar to that of CC (Table 5).

**Table 5:** Comparison of Na^+^ and K^+^ accumulation in different organs and transport in the trigeneric hybrid line T_3_ treated with different NaCl concentrations.

Added NaCl (mmol L^−1^)		Organs^a^		Ion content (mg g^−1^ DW)^b^				TS_K, Na_ c
				Na^+^	K^+^	Na^+^ + K^+^	K^+^/Na^+^	
0	R		0.80±0.04		17.19±0.67	17.99±0.71	21.55±0.19	–
	S		1.61±0.12		15.30±1.02	16.90±1.13	9.53±0.05	0.44±0.00
	L		2.15±0.15		38.86±1.96	41.01±2.11	18.09±0.38	1.90±0.03
50	R		4.51±0.35		23.49±1.80	28.00±2.14	5.21±0.01	–
	S		3.82±0.30		11.78±1.18	15.60±1.47	3.08±0.07	0.59±0.01
	L		15.71±1.43		25.18±1.86	40.89±3.27	1.61±0.03	0.52±0.02
100	R		3.11±0.10		31.26±1.25	34.37±1.35	10.06±0.10	–
	S		2.88±0.32		22.03±1.23	24.91±1.47	7.70±0.66	0.77±0.07
	L		19.94±1.31		25.56±1.78	45.50±3.09	1.28±0.01	0.17±0.01
150	R		4.26±0.30		31.61±1.81	35.86±2.10	7.43±0.10	–
	S		4.54±0.36		16.44±1.31	20.98±1.63	3.62±0.15	0.49±0.02
	L		23.46±1.73		24.35±2.08	47.81±3.80	1.04±0.01	0.29±0.01
200	R		4.10±0.33		23.43±1.65	27.53±1.98	5.72±0.09	–
	S		6.26±0.34		16.12±0.36	22.38±0.70	2.58±0.08	0.45±0.01
	L		28.70±1.72		24.43±1.91	53.13±3.62	0.85±0.02	0.33±0.02

a R, root; S, stem; L, leaf;

b the values represent mean ± SD; - represents no value;

c S_K, Na_ represents the selectivity ratio of K^+^ and Na^+^; TS_K, Na_ (transportation S_K, Na_) = (the value K^+^/Na^+^ of sink organ)/(the value K^+^/Na^+^ of source organ).

## Discussion

### Cross–compatibility between Intergeneric Hybrids and Selection of Parents for Multigeneric Hybridization

In the intergeneric crosses between *C. grandiflorum* and *A. vulgaris*, and *C. crassum* and *C. chinense*, although some pollen grains germinated normally on the stigma, the embryo only developed to the globular stage. Thus, serious reproductive barriers existed for these combinations and hybrids were only obtainable via embryo rescue [5], [19]. In the present study, the crosses between the two intergeneric hybrids showed a relatively weak pre-fertilization barrier, so fertilization was successful in most instances (Fig. 1). However, most of the hybrid embryos began to show signs of abortion in early stages of development; these severe post-fertilization barriers made it essential to employ embryo rescue to obtain the trigeneric hybrids (Fig. 2). The hybrids derived from the cross between *C. crassum* × *C. chinense* and *C. grandiflorum* × *A. vulgaris* carried a greater number of *Chrysanthemum* chromosomes, therefore their phenotypic characteristics were similar to their *Chrysanthemum* parents [5], [19]. When a chrysanthemum cultivar is crossed with a wild species with a different ploidy, the pollen germination behavior and embryo development pattern is different [23]. Therefore, interspecific hybridization of chrysanthemums, whether successful or not, is closely related to the ploidy of the parents; crosses are more likely to be successful when the ploidies of the parents are similar, which indicates that cross-compatibility requires a certain chromosomal or genomic balance [23], [24]. If the intergeneric hybrids, CA and CC, used in the present study are treated as a hexaploid and pentaploid *Chrysanthemum* species, respectively, the trigeneric hybridization is more akin to an interspecific cross between two chromosomally balanced species. Therefore, it is easy to understand the cross-compatibility and relatively high success rate of the embryo rescue procedure in this study. These results will aid with the selection of parents and/or bridge parents for multigeneric hybridization of chrysanthemums in the future.

### Combination of Multi-resistance for Chrysanthemum Improvement via Trigeneric Hybridization

The intergeneric hybrid of chrysanthemum and *A. vulgaris* (CA) showed much higher resistance to the aphid *Macrosiphoniella sanbourni* than its maternal parent ‘Zhongshanjingui’ in an inoculation test. This difference is because of the higher contents of monoterpenoids and sesquiterpenoids in the essential oil, and a higher density of trichomes and secretory glands on the leaves in CA [5]. In the present study, however, the trigeneric hybrid showed slightly lower aphid resistance than its maternal parent, the intergeneric hybrid CA (Table 3). Nevertheless, aphid resistance of the novel trigeneric hybrid was significantly higher than that of ‘Zhongshanjingui’, which is still a useful improvement for chrysanthemum germplasm innovation. More importantly, the trigeneric hybrid showed significantly enhanced tolerance to salinity (Fig. 5). As halophytes, *C. crassum* and *C. chinense* are adapted to salty environments and have developed high salt tolerance during their evolution [18], [19]. The results of the present study demonstrated that their intergeneric hybrid was also highly salt tolerant. In particular, the salt tolerance trait was well expressed in the trigeneric progeny. In addition, the intergeneric hybrid between chrysanthemum and *A. vulgaris* exhibits a superior rooting ability and enhanced leaf spot resistance compared with those of the chrysanthemum parent [5]. Although these two traits were not examined in the current study, it is reasonable to presume that they would be enhanced in the trigeneric hybrid and associated with the expression of *A. vulgaris* genes. Nevertheless, the present study demonstrated clearly that trigeneric hybridization is an effective means by which to combine multi-resistance for improvement of chrysanthemum, as has been shown for other crops such as oilseed rape and wheat [12], [25].

### Cytological Characteristics of the Trigeneric Hybrid and their Association with Resistance

GISH analysis revealed that the trigeneric hybrid T_3_ only carried nine chromosomes from *A. vulgaris*, half the number carried in the F_1_ hybrid of *C. grandiflorum* × *A. vulgaris* (Fig. 4). We considered that this is the likely reason for the reduction in aphid resistance in the trigeneric hybrid. In a pentaploid F_1_ hybrid chromosomes usually pair in several configurations, such as univalents, bivalents, and trivalents, and lagging chromosomes and bridge fragments are frequently observed at meiosis in pollen mother cells [10], [25]. The *Ph1* gene is well known to suppress pairing between unrelated (non-homologous) or less-related (homoeologous) chromosomes, but permits pairing between homologous partners in wheat [26]. In many allopolyploid flowering plants, however, the homoeologous chromosomes of different genomes are sufficiently similar to the extent that they are able to pair with one another [27]–[29]. Thus the occurrence of homoeologous chromosome pairing in interspecific hybrids is thought to be essential for gene transfer between species [26], [30]. In the present study, GISH revealed that the trigeneric hybrid obtained one set of chromosomes from each of *A. vulgaris* and *C. chinense* (Fig. 4). Given homoeologous chromosomes would pair and only euploid gametophytes were alive, the female parent CA would generate six types of megagametophytes of 9Cg9Av/9Cg9Cg9Av/9Cg9Cg/9Cg9Av9Av/9Cg9Cg9Cg/9Av9Av, where 9Cg and 9Av represent respectively nine chromosomes from *C. grandiflorum* and *A. vulgaris*. The male parent CC would generate two types of mircogametophytes of 9Ch9Ch9Ch/9Ch9Ch9Cr, where 9Ch and 9Cr represent nine chromosomes from *C. chinense* and *C. crassum*, respectively. In present study, GISH data inferred that the chromosomes in the hexaploid trigeneric T_3_ hybrid contribute to megagametophyte of 9Cg9Cg9Av and mircogametophyte of 9Ch9Ch9Cr. Thus, it is suggested that homoeologous chromosome pairing in the intergeneric hybrids occurred. However, this hypothesis needs further study by examination of meiotic behaviour in the intergeneric hybrids, and specifically chromosome pairing, orientation on the metaphase plate and subsequent separation at anaphase I. Nevertheless, the enhanced aphid resistance can be reasonably considered to be a contribution from *A. vulgaris*, whereas salinity tolerance is mainly attributable to *C. crassum* and *C. chinense*. These two species are difficult to distinguish with a GISH protocol because of their close phylogenetic relationship [22]. In addition, although GISH did not identify the chromosomes from *A. vulgaris* and *C. chinense* simultaneously, the other seven hybrid lines also showed morphological differences from their parents, thus it is worth detecting whether genes of *A. vulgaris* and *C. chinense* were incorporated into the genomes of *C. grandiflorum* and/or *C. crassum*, or simply represent heterozygous segregation among interspecific hybrid progeny of *Chrysanthemum* [31].

### Salt Tolerance Mechanisms in Chrysanthemum and its Wild Relatives

Salinity involves ionic stress, osmotic stress, and secondary stresses such as oxidative stress and nutritional imbalances for plants [32]. To cope with the detrimental effects of salt stress, plants that grow in saline environments have evolved various adaptive strategies, including morphological, anatomical and biochemical adaptations [33]. Some of the biochemical strategies include selective buildup or exclusion of salt ions, control of ion uptake by roots and transport into leaves, ion compartmentalization, synthesis of compatible osmolytes, and alterations in the photosynthetic pathway [34]. Consistent with increasing NaCl concentration, in the present study the test plants adsorbed more ions into the roots. However, different lines show entirely different responses in ion transportation and distribution, and thus compartmentalize essential ions in different tissues [35]. In the maternal parent, CA, large quantities of Na^+^ and K^+^ ions were transported to the leaves, which severely damaged the photosynthetic apparatus (Table 4; Fig. 6). In contrast, in the paternal parent, CC, and trigeneric hybrid a much higher quantity of ions were stored in stems to minimize their accumulation in leaves and thereby protect chloroplast development and chloroplast functioning (Tables 4, 5; Figs. 7, 8). Thus, it can be concluded that ion compartmentalization and selective transportation to protect the photosynthetic apparatus is an important salt tolerance mechanism in chrysanthemum and its wild relative species.

## Materials and Methods

### Plant Materials, Growth Conditions and Artificial Pollinations

*Chrysanthemum grandiflorum* ‘Zhongshanjingui’ is hexaploid (2*n* = 6*x* = 54), *A. vulgaris* ‘Variegata’ is tetraploid (2*n* = 4*x* = 36), and their F_1_ hybrids are pentaploid (2*n* = 5*x* = 45) [5]. *Chrysanthemum crassum* is decaploid (2*n* = 10*x* = 90), *Crossostephium chinense* is diploid (2*n* = 2*x* = 18), and their F_1_ hybrids are hexaploid (2*n* = 6*x* = 54) [19]. The bigeneric F_1_ hybrid plants (*C. grandiflorum* ‘Zhongshanjingui’ × *A. vulgaris* ‘Variegata’) (CA) and (*C. crassum* × *C. chinense*) (CC) were cultivated in a greenhouse (day/night temperatures 25/18°C, photoperiod 14 h, light intensity 45–50 µmol m^−2^ s^−1^, and relative humidity 70–75%) at the Chrysanthemum Germplasm Resource Preservation Centre, Nanjing Agricultural University, China (32°05′N, 118°8′E, 58 m altitude). A total of 150 maternal inflorescences were emasculated and covered with paper bags at the stage before stigmas were visible, and artificial pollination was performed using the method described by Deng et al. [6].

### Pollen–pistil Interaction, Embryo Development and Rescue

Examination of pollen germination on stigmas followed the method of Deng et al. [6] with minor revision. For observation under a fluorescence microscope, five inflorescences (containing ∼120 pistils) at each time point were fixed in FAA solution (5∶5∶90 formalin: acetic acid: 70% ethanol, v/v) at 1, 2, 4, 8, 12, 24 and 48 h after pollination (HAP). For examination with a scanning electron microscope, three inflorescences were fixed in 2.5% glutaraldehyde (0.1 mol·L^−1^ phosphate buffer, pH 7.2) at 1, 2, 4, 8, 12 and 24 HAP.

To examine embryo development, five inflorescences per time point were collected at 2, 4, 6, 8, 10, 12, 15 and 18 days after pollination (DAP) and fixed in FAA. The samples were prepared for paraffin section following the procedures of Deng et al. [36]. The other pollinated plump ovaries were removed from the female flowers at 10–15 DAP and were surface-sterilized and washed to rescue embryos, following Deng et al. [7].

### Investigation of Morphological Characteristics for Preliminary Hybridity Test

Morphological traits, consisting of the onset of flowering, plant height, crown width, inflorescence characteristics and leaf shape, of the putative trigeneric hybrids were compared with those of the parents. Inflorescence characteristics were quantified by the central disc diameter (DD), inflorescence diameter (ID) and the ratio of ID/DD, and the numbers of tubular disc florets (NT) and ligulate ray florets (NL) and their ratio (NT/NL); 10 inflorescences were measured for each trait. Description of leaf shape comprised a combination of length, width, and the length/width ratio, measured on the fifth leaf below the shoot apex and recorded from a sample of 10 leaves [7].

### Chromosome Number and GISH Analysis for Hybridity Test

Determination of the chromosome number and GISH employed a method based on that of Deng et al. [7] using young root tips. For multicolor GISH, the preparations were stained with 4′,6-diamidino-2-phenylindole (DAPI; blue fluorescence), and with probes for genomic DNA of the parental species *C. chinense* and *A. vulgaris*, namely fluorescein-12-dUTP (Roche, Berlin, Germany; green fluorescence) and Cy™-3-dUTP (GE Healthcare, London, UK; red fluorescence), respectively.

### Evaluation of Aphid Resistance

Aphid resistance was evaluated in confirmed trigeneric hybrids, both parents and the chrysanthemum cultivar ‘Zhongshanjingui’ (as a control) in accordance with the methods described by Deng et al. [5] with minor revision. The number of aphids was measured at 21 d post inoculation, and the multiplication rate (MR) of aphids at this time point was used to classify the resistance level: plants with an MR of <4 were considered to be highly resistant, those with an MR in the range 4 to 8 were moderately resistant, and those with an MR >8 were weakly resistant. The aphid resistance of the trigeneric progeny was compared with the control and its parents by calculation of the inhibition ratio (IR) using the formulas IR_Zh_ = (MR_Zh_ – MR_i_)/MR_Zh_, IR_CA_ = (MR_CA_ – MR_i_)/MR_CA_ and IR_CC_ = (MR_CC_ – MR_i_)/MR_CC_, respectively, where MR_Zh_, MR_CA_, MR_CC_ and MR_i_ represent the corresponding MR of ‘Zhongshanjingui’, CA, CC and the progeny.

### Evaluation of Salt Tolerance

For evaluation of salt tolerance, a set of 30 seedlings with a developed root system from each trigeneric hybrid line and both parents were cultured in aerated Hoagland nutrient solution in 23.4 L plastic boxes under greenhouse conditions (22±3°C, a photoperiod of 16 h, light intensity 45–50 µmol m^−2^ s^−1^ and relative humidity 75%), as described by Guan et al. [17]. Each plant was potted in a 300 ml plastic cup that contained quartzite gravel. After a one-week adaptive period, 20 plants per treatment were selected and watered with Hoagland solution supplemented with 0, 50, 100, 150 or 200 mmol L^−1^ NaCl. The percentage of injured plants and leaves was recorded from 3 to 7 days after stress (DAS). The roots, stems, and fifth leaf from the shoot apex of each plant were collected for measurement of the contents of Na^+^ and K^+^ ions at 7 DAS. The Na^+^ and K^+^ ions were extracted following the method of Wang and Zhao [37] and quantified with an inductive coupling plasma emission spectrograph (Optimal 2100DV, PerkinElmer, Boston, USA). The transportation selectivity ratio of K^+^ and Na^+^ (TS_K,Na_) was calculated with the formula: TS_K,Na_ = (the value K^+^/Na^+^ of sink organ)/(the value K^+^/Na^+^ of source organ). Observation of the ultrastructure of leaf mesophyll cells was conducted with a transmission electron microscope (TEM, H7650, Hitachi, Tokyo, Japan) using methods described by Deng et al. [38].

### Statistical Analysis

All data were analysed by one-way analysis of variance using the software package SPSS 11.5 for Windows, and Duncan’s multiple range test was employed to detect differences between means (with a level of significance of 0.05).
